# Genomic Evidence for Convergent Molecular Adaptation in Electric Fishes

**DOI:** 10.1093/gbe/evab038

**Published:** 2021-02-26

**Authors:** Ying Wang, Liandong Yang

**Affiliations:** 1 College of Life Sciences, Jianghan University, Wuhan, 430056, China; 2 State Key Laboratory of Freshwater Ecology and Biotechnology, Institute of Hydrobiology, Chinese Academy of Sciences, Wuhan, 430072, China

**Keywords:** convergent evolution, electric fishes, *scn4aa*, transcriptome

## Abstract

Fishes have independently evolved electric organs (EOs) at least six times, and the electric fields are used for communication, defense, and predation. However, the genetic basis of convergent evolution of EOs remains unclear. In this study, we conducted comparative genomic analyses to detect genes showing signatures of positive selection and convergent substitutions in electric fishes from three independent lineages (Mormyroidea, Siluriformes, and Gymnotiformes). Analysis of 4,657 orthologs between electric fishes and their corresponding control groups identified consistent evidence for accelerated evolution in electric fish lineages. A total of 702 positively selected genes (PSGs) were identified in electric fishes, and many of these genes corresponded to cell membrane structure, ion channels, and transmembrane transporter activity. Comparative genomic analyses revealed that widespread convergent amino acid substitutions occurred along the electric fish lineages. The overlap of convergent genes and PSGs was identified as adaptive convergence, and a subset of genes was putatively associated with electrical and muscular activities, especially *scn4aa* (a voltage-gated sodium channel gene). Our results provide hints to the genetic basis for the independent evolution of EOs during millions of years of evolution.

Significance statement

Fishes have independently evolved electric organs at least six times, and therefore this is a model system for understanding the molecular mechanisms of convergent evolution. In this study, we conducted comparative genomic analyses to detect genes showing signatures of positive selection and convergent substitutions in electric fishes from three independent lineages (Mormyroidea, Siluriformes, and Gymnotiformes). We found evidence of convergent evolution between these electric fishes, including an accelerated rate of protein evolution, positive selection, and convergent substitutions. Importantly, we found that the *scn4aa* gene showed convergence between these electric fish, thus providing evidence for convergent evolution.

## Introduction

Convergent evolution highlights how selective pressures can shape distantly related species in similar ways. This is often referred to as adaptive phenotypic convergence (Christin et al. 2010; Bittleston et al. 2016). Adaptive phenotypic convergence often results from genetic convergence, ranging from the same metabolic and regulatory pathways to identical amino acid substitutions in distant lineages (Christin et al. 2010; Stern 2013). Recent technological advances in genome and transcriptome analyses have opened up new opportunities to explore the genetic mechanisms underlying adaptive convergence (Parker et al. 2013; Foote et al. 2015; Zhou et al. 2015; Davies et al. 2018). For example, convergent amino acid changes have occurred across the genome during the independent evolution of echolocating cetaceans and bats (Parker et al. 2013). Similarly, convergent amino acid substitutions and positive selection associated with an aquatic lifestyle in three distinct groups of marine mammals were revealed by comparative genomic analyses (Foote et al. 2015; Zhou et al. 2015). These results showed that adaptive molecular convergence associated with the aquatic lifestyle is relatively rare, with higher levels of convergent amino acid substitutions detected in a control set of terrestrial sister taxa compared to the marine mammals (Foote et al. 2015).

Among the more than 30,000 fish species in the world, about 350 fishes have specialized electric organs (EOs). Therefore, such fishes are known as electric fishes. Electric fishes are mainly distributed in the ocean and freshwater rivers of Africa and South America, and they are classified as strongly electric fishes and weakly electric fishes (Nelson 2011). Strongly electric fishes generate intermittent discharge pulses for predation and defense, while weakly electric fishes generate less than a single volt of charge for sensing and communication. The reason why electric fishes possess the ability to generate electric fields by EOs is that many electric fishes are nocturnal and inhabit murky waters. EOs are made up of electrocytes, a unique vertebrate trait that has independently arisen at least six times (Gallant et al. 2014). The major groups of electric fishes span five orders of fish species, the Torpediniformes (the marine electric rays) and Rajiformes (skates), Osteoglossiformes (the African freshwater Mormyridae and Gymnarchidae), Gymnotiformes (the South American knifefishes), Siluriformes (several catfish species), and Perciformes (several marine stargazers) (Gallant et al. 2014; Lamanna et al. 2015). The electric fish lineages over broad taxonomic scales provide a valuable model for convergent evolution. Nevertheless, explorations of convergent evolution in electric fish have only focused on gene expression or some important genes (e.g., sodium channel genes) (Zakon et al. 2006; Gallant et al. 2014). However, the underlying genetic architecture of the EO from the perspective of molecular convergent evolution remains unresolved.

In this study, transcriptome-wide coding DNA sequences of nine species from three independent electric fish lineages (Mormyroidea, Siluriformes, and Gymnotiformes) were used in an analysis of convergent evolution. To explore the extent to which convergent molecular changes have occurred in EOs of three electric fish lineages, we first performed an evolutionary rate analysis on focal branches from three independent electric fish lineages, and we conducted positive selection and GO functional enrichment analyses. Second, to detect molecular convergence patterns, we identified convergent amino acid substitutions of electric fish lineages and compared PSG sets in terms of orthology and functional annotations. Finally, we made functional analyses for some potential genes associated with electrogenesis.

## Materials and Methods

### Bioinformatic Data Sources and Transcriptome Assembly

**Fig. 1.**
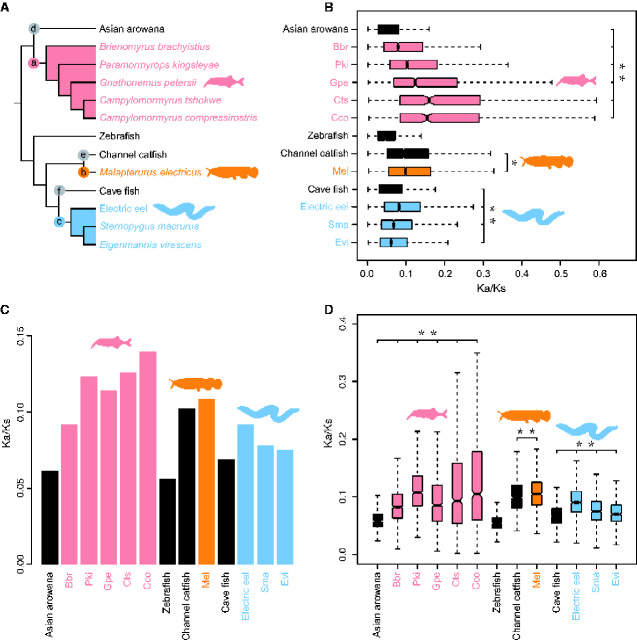
Species tree topology used for evolutionary analyses (A), and branch specific Ka/Ks ratios obtained from different data sets (B–D). Each of the three electric fish clades was labeled as follows: a-Mormyroidea, b-*Malapterurus electricus*, and c-Gymnotiformes; and nonelectric taxa used for comparisons are labeled as follows: d-Asian arowana, e-Channel catfish, and f-Cave fish. Images of representative electric fishes are provided (A). The Ka/Ks ratios for each terminal branch were calculated from all 4,657 orthologs (B), concatenated all orthologs (C), and 1,000 concatenated alignments constructed from ten randomly chosen orthologs (D).

Genome-wide coding DNA sequences (CDSs) from 13 fish species were obtained, including 9 electric fish species from 3 independent lineages (Mormyroidea, Siluriformes, and Gymnotiformes) (fig. 1*A*): (1) five fish species from Mormyroidea: *Brienomyrus brachyistius*, *Paramormyrops kingsleyae*, *Gnathonemus petersii*, *Campylomormyrus tshokwe*, and *Campylomormyrus compressirostris*; (2) one fish species from Siluriformes: *Malapterurus electricus*; and (3) three fish species from Gymnotiformes: *Electrophorus electricus*, *Sternopygus macrurus*, and *Eigenmannia virescens*. Raw reads of these nine focal electric fish transcriptomes were retrieved from the Sequence Read Archive of NCBI. Detailed information concerning these transcriptome data is listed in supplementary table S1 (Gallant et al. 2014, 2017; Lamanna et al. 2014, 2015). The above raw RNA-seq reads for each species were filtered using TrimGalore (Barbraham, Bioinformatics) and assembled using Trinity (Grabherr et al. 2011) according to methods detailed in our previous studies (Wang et al. 2015b; Yang et al. 2015).

### Ortholog Identification and Alignment

One-to-one orthologs between Zebrafish and Cave fish were retrieved from Ensembl (Release 86) using the BioMart tool (Vilella et al. 2009). CDSs of Channel catfish and Asian Arowana genomes were obtained from NCBI (Bian et al. 2016; Chen et al. 2016). CDSs of nine other electric fishes were retrieved from their assembled transcriptomes. Putative single-copy orthologs between zebrafish and channel catfish, Asian Arowana, and nine other electric fish transcriptomes were identified using the reciprocal BLASTX best-hit method with an *E* value cutoff of 1 × e^−5^. When more than one alternatively spliced transcript was identified, the longest was retained. Each orthologous gene set from 13 species was aligned using MAFFT version 7.310 (Katoh and Standley 2013) at the protein level, and coding sequences were generated and aligned with pal2nal version 14 (Suyama et al. 2006), and then filtered using the Gblocks program with the parameter “-t = c” (Castresana 2000). Finally, a total of 4,657 orthologs longer than 90 bp were generated for further analyses.

### Genome-Wide Evolution Analysis

We constructed all orthologs from each fish species in the same order to generate concatenated alignments. Concatenated alignments were used to build a phylogenetic tree of 13 teleosts using RAxML 7.0.3 (Stamatakis 2006). The process of building the phylogenetic tree followed Wang et al. (2015b), including an optimal partition scheme and model choosing, and the parameters of the phylogenetic tree were reconstructed. The lineage-specific Ka/Ks (nonsynonymous–synonymous) substitution ratios of each branch were computed by the codeml program of the PAML package (Yang 2007). Three computing strategies using each ortholog, concatenated orthologs, and randomly chosen orthologs with replicates were calculated as in Wang et al. (2015b). The parameters of a free ratio model were set and the Ka/Ks values were filtered following Wang et al. (2015b). The Ka/Ks ratio value was designated as the evolutionary rate. The evolutionary rate of the electric fish branch was compared with that of the nonelectric fish branch to test biological significance through the Wilcoxon rank-sum test.

### The Branch-Site Test for Positive Selection

To identify genes involved with EOs, the branch-site test (model A) implemented in codeml from PAML package (Yang 2007) was used to test for positive selection on individual codons along the lineage leading to each of the three independent origins of electric fishes. In this test, an alternative model allowing positive selection on the foreground lineage (branches a–c, fig. 1*A*) was contrasted with a null model that did not allow such positive selection using a likelihood-ratio test (Zhang et al. 2005). To filter out genes that are always under positive selection and to gain a better understanding of which genes that actually could be tied to EOs, repeated selection pressure tests were performed on each of three corresponding close nonelectric fishes (Asian arowana, Cave fish, and Channel catfish) (branches d–f, fig. 1*A*). The parameters of the improved branch-site model were chosen, and whether positive selection occurred along each branch was estimated according to Wang et al. (2015b). We used the Benjamini-Hochberg method to perform multiple tests, and a false discovery rate (FDR) threshold of 0.05 was set. Intersections of positively selected genes (PSGs) between electric fish branch pairs were visualized using UpSet (Lex et al. 2014), and significance testing was assessed using supertest from the SuperExactTest package(Wang et al. 2015a).

### Identification of Convergence at the Molecular Level

To keep the number of electric fishes comparable to the number of nonelectric fishes, *P. kingsleyae*, *E. electricus*, and *M. electricus*were were chosen as representatives for each electric fish lineage. The genomic data of *P. kingsleyae* and *E. electricus* were retrieved from the EFISHGENOMICS web portal *(*http://efishgenomics.integrativebiology.msu.edu*).* The genomic data of *M. electricus* and other species (Zebrafish, Cave fish, Channel catfish, and Asian Arowana) were the same as in the former ortholog identification and alignment section. A total of 6,449 orthologs were identified among these seven teleosts. The ancestral amino acid sequences were reconstructed for these 6,449 orthologs using the codeml program in the PAML package (version 4.7) (Yang 2007). Convergent sites were identified if an amino acid residue changed at the same loci in the three electric fish lineages and was different from that of their respective most recent common ancestor but identical in the three electric fish lineages. Specifically, the rules used were as follows: (i) The amino acid residues of all three electric fish branches were identical; (ii) amino acid change occurred between the extant *P. kingsleyae* lineage and its ancestral node shared with the Asian Arowana branch; (iii) amino acid change occurred between the extant *M. electricus* branch and its ancestral node shared with the channel catfish branch; and (iv) amino acid change occurred between the extant *M. electricus* and its ancestral node shared with cave fish branch.

### GO Enrichment Tests

To assess functional enrichment of PSGs and convergent genes, we used Fisher’s exact test in combination with the “classic,” “elim,” and “weight” algorithms implemented in topGO v.2.32 of Bioconductor (Gentleman et al. 2004; Alexa et al. 2006), to test for overrepresented GO-terms within the three domains: biological process (BP), molecular function (MF), and cellular component (CC). To avoid small sets of genes precluding meaningful tests of enrichment, we used the gene sets based on PSGs with *P* < 0.05. The “classic” algorithm deals with each GO term independently without considering the GO-hierarchy; the “elim” algorithm discards genes that have already been mapped to significant child terms when traversing the GO-graph bottom-up, and the “weight” algorithm determines which GO-terms best represent the gene based on a weighting scheme in view of the enrichment scores of the neighboring GO-terms (Alexa et al. 2006). Previous studies using simulations have shown that the “elim” and “weight” algorithms are prone to be more conservative than the “classic” algorithm, and it is recommended to use the “elim” algorithm to detect important areas in the graph due to its simplicity (Alexa et al. 2006). The genes from the zebrafish genome were used as the background gene universe in all gene set enrichment analyses. According to the recommendations of the authors, we did not use FDR adjustment of the “classic” algorithm values to avoid being overly conservative.

## Results

### Final Alignment Construction and Phylogeny

We obtained genome-wide coding sequences from 13 fish species, including three independent electric fish lineages (Mormyroidea, Siluriformes, and Gymnotiformes, 9 fish species in total) and their nonelectric fish sister taxa (4 fish species) (supplementary table S1). A total of 4,657 orthologous gene groups of 13 fish species were generated for alignment construction and were used for the subsequent analyses. A phylogenetic tree of 13 fish species was constructed based on the above concatenated 4,657 orthologs (fig. 1*A*).

### Accelerated Evolution in the Electric Fish Lineages

Across all 4,657 gene orthologs, the lineage-specific Ka/Ks ratios (the ratios of the number of nonsynonymous substitutions per nonsynonymoussite [Ka] to the number of synonymous substitutions per synonymous site [Ks]) of each ortholog across each branch (codeml; a free ratio model) showed that all electric fish lineages had significantly higher ω (Ka/Ks) ratios than their paired control nonelectric fishes [*P *<* *2.2e-16 for five mormyroids vs. Asian arowana, *P *=* *0.01093 for the electric catfish from Africa (*M. electricus*) vs. Channel catfish, and *P *=* *2.102e-11 for three Gymnotiformes vs. Cave fish] (fig. 1*B*). Moreover, we calculated the Ka/Ks ratio of a concatenated alignment of 4,657 orthologs for each branch and the Ka/Ks ratio for each branch for 1,000 concatenated alignments constructed from 10 randomly chosen orthologs. Intriguingly, using the above both datasets, we also found that all electric fish lineages exhibited significantly elevated Ka/Ks ratios than their paired control nonelectric fishes [*P *<* *2.2e-16 for five mormyroidsvs Asian arowana, *P *=* *0.0001918 for the electric catfish from Africa (*M. electricus*) vs. Channel catfish, and *P *=* *2.446e-05 for three Gymnotiformes vs. Cave fish] (fig. 1*C* and 1*D*). These results suggested that all electric fish lineages exhibited more rapid genome-wide evolution than their control nonelectric fishes.

### Genes under Positive Selection and Their GO Function Enrichment in Electric Fishes

**Fig. 2.**
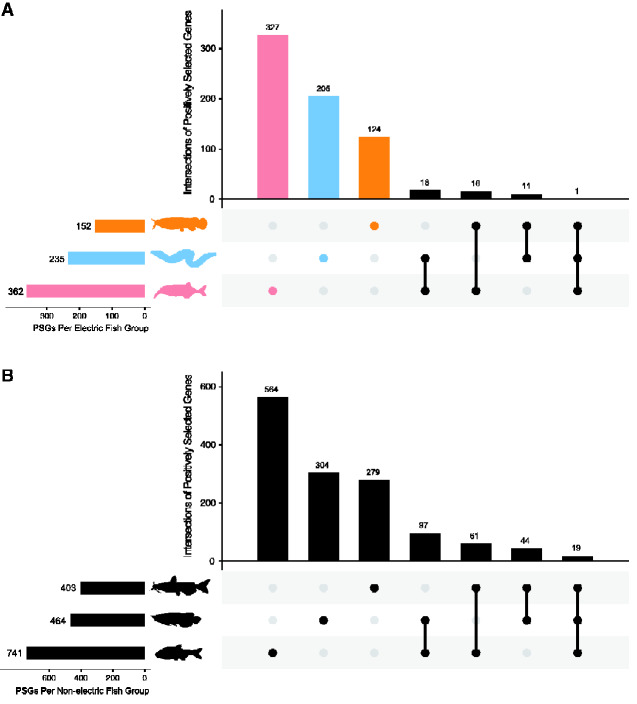
(A) UpSet plot of PSGs identified in three electric fish lineages. (B) UpSet plots of the PSGs identified in corresponding nonelectric fish lineages. The plot in the left corner indicates total number of PSGs. The main plot shows the number of unique PSGs and the connected dots show the PSG intersections/overlaps between species.

Based on 4,657 orthologous genes, we performed branch-site model tests to detect PSGs in three individual electric fish branches. Three different likelihood ratio tests were performed on the ancestral branch of five mormyroids from Africa, the ancestral branch of three species of Gymnotiformes, and the terminal branch of *M. electricus* (represented by a single species). We identified 362 PSGs in Mormyroidea, 152 PSGs in *M. electricus* and 235 PSGs in Gymnotiformes (fig. 2*A*, table 1, and supplementary tables S2–S4). In addition, we conducted complementary analyses in three nonelectric reference species, yielding 464 PSGs in Asian arowana, 403 PSGs in channel catfish and 741 PSGs in cave fish (fig. 2*B*, table 1, and supplementary tables S5–S7).

**Table 1:** Number of PSGs detected by the branch-site test, as well as corresponding topGO results.

Branch	Lineage tested	**PSGs *P* < 0.05** **[FDR *P* < 0.05]**	Enriched GO-terms a BP, CC, MF
(a) Electric fish	Mormyroidea	362 [13]	356 (225), 114 (65), 137 (116)
(b) Electric fish	*Malapterurus electricus*	152 [15]	216 (172), 44 (39), 74 (57)
(c) Electric fish	Gymnotiformes	235 [5]	341 (226), 58 (40), 141 (106)
(d) NonElectric fish	Asian arowana	464 [49]	321 (228), 122 (86), 127 (105)
(e) NonElectric fish	Channel catfish	403 [105]	403 (261), 120 (89), 117 (104)
(f) NonElectric fish	Cave fish	741 [387]	589 (381), 127 (85), 148 (112)

a Numbers of enriched GO terms (*P* < 0.05) reported are those detected with Fisher’s exact test using the classic algorithm, and numbers in brackets are those remaining significant under the elim algorithm.

(a), Mormyroidea; (b), Malapteruridae; (c), Gymnotiformes; (d), Asian arowana; (e), Channel catfish; (f), Cave fish.

To evaluate whether positive selection acted on the same genes, we compared PSGs of each of three electric fish lineages. We found that 46 PSGs were shared in at least two electric fish lineages without a FDR adjustment (fig. 2*A*). PSG intersections occurring between each pair of three electric fish lineages were greater than the expected overlap (supplementary table S8) using the supertest function from the SuperExactTest package (Wang et al. 2015a). Among these 46 PSGs, one was identified in all three electric fish lineages (fig. 2*A*). Of the PSGs shared by both *M. electricus* and Gymnotiformes electric fish lineages, *camk2b1* (calcium-dependent protein kinase II beta 1) is involved with Ca^2+^release from the sarcoplasmic reticulum during contraction and excitation-contraction coupling (Shakiryanova et al. 2007; Boczek et al. 2016). The membrane-spanning protein *tmem206* (transmembrane protein 206), as a crucial ASOR component, mediates moderately different ICl, H currents (Ullrich et al. 2019). The myosin, light chain kinase 5 (*mylk5*) regulates the sarcomeres in muscle contraction during the production of electricity and involves in IGF signaling pathway genes (Gallant et al. 2014). Of the PSGs shared by both Mormyroidea and Gymnotiformes electric fish lineages, *ckmt2b*, creatine kinase, mitochondrial 2 b (sarcomeric) is expressed in striated muscles (Porter et al. 2003). Of the PSGs shared by both Mormyroidea and *M. electricus*, *kcnip4* (potassium voltage-gated channel interacting protein 4), which is involved in potassium channels, was identified as under positive selection in this study. PSGs in each of the three electric fish lineages were used in GO functional enrichment analyses respectively across three domains, including MF, CC, and BP (table 1 and supplementary tables S9–S11). To filter out the enriched GO-terms unlikely to be involved with EO discharge behavior, we also conducted a GO functional enrichment analysis in each of three corresponding close nonelectric fishes (supplementary tables S12–S14). Enriched GO terms across domains were counted, and Mormyroidea possessed the most enriched GO terms among the three electric fish lineages. Several enriched GO terms were potentially associated with producing electricity, including regulation of cardiac muscle contraction by calcium ion signaling, calcium channel regulator activity, and regulation of muscle contraction.

### Widespread Convergent Amino Acid Substitutions in Electric Fishes

**Fig. 3.**
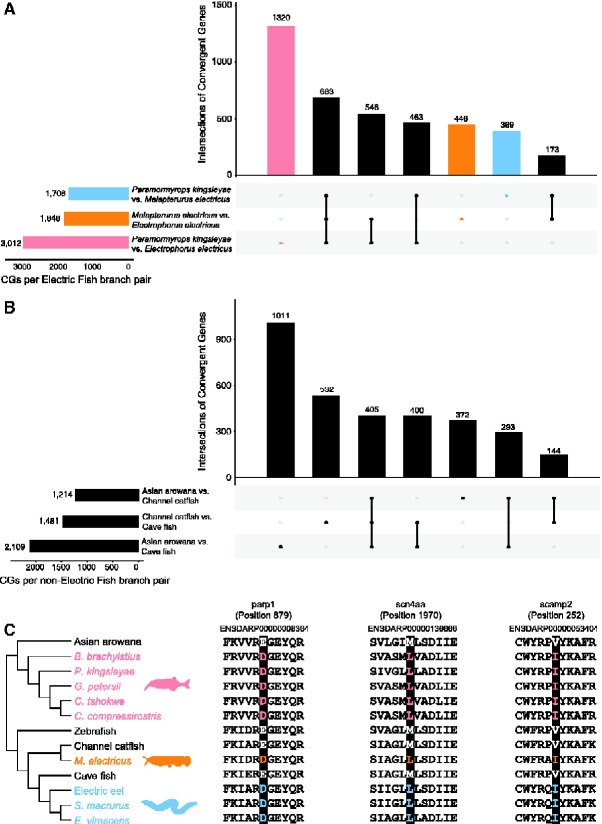
(A) UpSet plot of convergent genes identified between pairs of electric fish lineages. The plot in the left corner indicates total numbers of convergent genes. The main plot indicates the number of unique convergent genes and the connected dots indicate the convergent intersections between branch pairs. (B) UpSet plot of convergent genes identified between pairs of nonelectric fish lineages. (C) Comparison of shared convergent amino acid replacements in scn4aa, parp1, and scamp2 among nine electric fish species and four nonelectric fish species.

To identify convergent sites in the three electric fish lineages, we reconstructed ancestral protein sequences for 6,449 orthologs based on the phylogenetic tree of 7 teleosts, including one representative for each electric fish lineage. We identified a total of 4,019 convergent genes among at least 2 electric fish lineages. A total of 235 convergent genes were identified among all three electric fish lineages. The highest number of convergent genes (3,012) was found between *P. kingsleyae* and *E. electricus*, followed by *M. electricus* and *E. electricus* (1,848), and *P. kingsleyae* and *M. electricus* (1,708; fig. 3*A*, table 2, and supplementary tables S15–S18). For comparison, 3,155 convergent genes were identified along three nonelectric fish control groups (fig. 3*B*, table 2, and supplementary tables S19–S22), suggesting that there are widespread convergent amino acid substitutions in electric fishes. GO enrichment tests of convergent genes (supplementary tables S23–S26) showed that “voltage-gated sodium channel complex” (CC) was significantly enriched in the combined three electric fish lineages while none occurred in the nonelectric fish comparisons (supplementary tables S27–S30). Several convergent genes had functional associations with electrical and muscular activity and exhibited convergent amino acid substitutions in the three independent electric fish lineages (fig. 3*C*). For example, *scn4aa* (sodium channel, voltage-gated, type IV, alpha, and a) is involved with ion channel transport and is differentially expressed between EO and SM (skeletal muscle). This gene apparently played a vital role in the evolution of electric communication (Arnegard et al. 2010; Lamanna et al. 2015). The gene *parp1* [poly (ADP-ribose) polymerase 1] exhibits differentiation-associated down-regulation in myoblasts that serves to increase their resistance to muscle contraction (Olah et al. 2015). *net1* (neuroepithelial cell transforming 1) may play a role in repairing DNA damage after ionizing radiation (Srougi and Burridge 2011). *scamp2* (secretory carrier membrane protein 2) regulates membrane dynamics of neuronal and neuroendocrine signaling (Zheng et al. 2014). *mybpc2a* (myosin binding protein C, fast type a) is down-regulated in the EO and is associated with striated muscle contraction (Lamanna et al. 2015).

**Table 2:** Number of convergent genes, as well as corresponding topGO results.

Branch compared	Lineages tested	No. of convergent genes	Enriched GO-terms a BP, CC, MF
*Paramormyrops kingsleyae*, *Malapterurus electricus*, *Electrophorus electricus*	Electric, Electric, Electric	235	354 (228), 100 (71), 148 (108)
*Paramormyrops kingsleyae*, *Malapterurus electricus*	Electric, Electric	1,708	689 (391), 125 (77), 216 (143)
*Paramormyrops kingsleyae*, *Electrophorus electricus*	Electric, Electric	3,012	983 (651), 185 (108), 258 (195)
*Malapterurus electricus, Electrophorus electricus*	Electric, Electric	1,848	735 (409), 179 (112), 244 (153)
Asian arowana, Channel catfish, Cave fish	NonElectric, NonElectric, NonElectric	131	229 (149), 39 (25), 89 (73)
Asian arowana, Channel catfish	NonElectric, NonElectric	1,214	669 (458), 120 (87), 243 (193)
Asian arowana, Cave fish	NonElectric, NonElectric	2109	877 (505), 131 (98), 231 (158)
Channel catfish, Cave fish	NonElectric, NonElectric	1481	692 (372), 134 (85), 225 (154)

a Numbers of enriched GO terms (*P* < 0.05) reported are those detected with Fisher’s exact test using the classic algorithm, and numbers in brackets are those remaining significant under the elim algorithm.

**Fig. 4.**
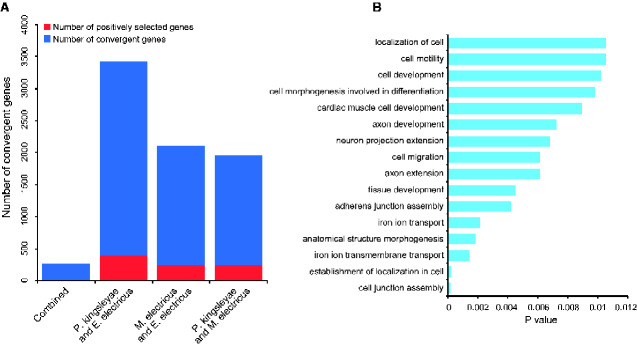
Statistics of adaptively convergent genes and functional enrichment. (A) The intersections of convergent genes and PSGs that occurred along the branches of at least two electric fish lineages. Convergent genes that occurred in PSGs are shaded red. (B) Functional enrichment of adaptively convergent genes.

The convergent genes were compared with the above identified PSGs. The intersection between these two sets was inferred as adaptively convergent genes. Twenty-four of 235 convergent genes have apparently evolved under positive selection in all three electric fish lineages. For example, *fam65a* (family with sequence similarity 65, member A), *tln1* (talin 1), *eea1* (early endosome antigen 1). A total of 249 of 1,708 convergent genes have apparently undergone positive selection in *P. kingsleyae* and *M. electricus*. In addition, 406 of 3,012 convergent genes have undergone positive selection in *P. kingsleyae* and *E. electricus*, whereas 257 of 1,848 convergent genes have apparently undergone positive selection in *M. electricus* and *E. electricus* (fig. 4*A*). The enriched network of adaptively convergent genes is shown in figure 4*B*.

## Discussion

With multi-omics data becoming widely available, comparative genomic analyses have been extensively used to explore the genetic basis underlying adaptive convergence (Foote et al. 2015; Hu et al. 2017; Hao et al. 2019). In this study, we attempted to investigate the signatures of convergent evolution across three major lineages of electric fishes. On the one hand, we collected the latest data and widened the taxon samples. On the other hand, we combined analyses of positive selection and loci mutation to investigate the extent to which molecular convergence has occurred in electric fishes from three independent lineages (Mormyroidea, Siluriformes, and Gymnotiformes). Although the genetic basis of these three lineages has been studied previously (Gallant et al. 2014; Lamanna et al. 2015; Liu et al. 2019), only patterns of gene expression were analyzed in those studies and one or two representative species of each lineage were included. Primarily, studies of convergent evolution in electric fish lineages have been restricted to gene expression changes in transcription factors, developmental pathways, and biological functions (Gallant et al. 2012, 2014; Lamanna et al. 2015). However, no evidence was found from the selective pressures or adaptive convergent amino acid substitutions of protein-coding genes. Therefore, a comparative genomic analysis was performed to detect orthologous genes showing signatures of positive selection and convergent amino acid substitutions in electric fishes from the three independent lineages (Mormyroidea, Siluriformes, and Gymnotiformes).

Electric fishes live in turbid rivers with low oxygen levels and sandy or muddy bottoms, and therefore the ability to produce electricity is useful for communication, stunning prey, and discouraging potential predators (Nelson 2011). Inhabiting such an ecological niche, these fishes might be expected to undergo adaptive evolution for electric signal sensing and communication. The evolutionary rate of genetic change is an important indicator of the interaction between the environment and genotypes of organisms. Within unchallenging and stable environments, species often display slow evolutionary rates, for example in the coelacanth (Amemiya et al. 2013). Nevertheless, in harsh environments, species often meet with hostile conditions and must adapt much faster, showing accelerated evolutionary rates (Wang et al. 2015b; Yang et al. 2015). In this study, faster evolutionary rates for protein-coding genes were found in all three independent electric fish lineages, suggesting active interaction between electric fishes with their environment. However, it should be acknowledged that teleosts experienced a specific whole genome duplication (Taylor et al. 2003) that may have influenced ortholog identification and evolutionary rate analyses.

Our positive selection analysis based on 4,657 orthologous genes across three electric fish lineages identified 702 PSGs. Of these, 46 PSGs (6.5%) were shared in at least two electric fish lineages, and one was identified in all three electric fish lineages without a FDR adjustment. In comparison, 221 PSGs (16.2%) were found in at least two nonelectric fish lineages. A similar phenomenon in which the number of PSGs in Arctic lineages was less than in nonArctic lineages was also identified in the Arctic Brassicaceae (Birkeland et al. 2020). Nevertheless, for electric fish lineages, some PSGs were related to the process of electrogenesis; for example, the *camk2b1* gene is involved with Ca^2+^ release from sarcoplasmic reticulum during contraction and excitation contraction coupling (Shakiryanova et al. 2007; Boczek et al. 2016), and in gene expression changes of sarcomeres (Gallant et al. 2014). The *tmem206* (transmembrane protein 206) gene encodes a membrane-spanning protein mediating different ICl, H currents (Ullrich et al. 2019). GO enrichment analyses of PSGs provided further insights into several functional classes associated with producing electricity, including transmembrane transport and organic substance transport.

With respect to convergence at the sequence level, we identified many protein-coding genes containing putative convergent amino acid replacements in electric fishes than in nonelectric fishes. This result was consistent with a previous study focusing on gene expression convergence in electric fishes (Gallant et al. 2014), thus demonstrating widespread molecular convergence in electric fishes. Furthermore, we found several convergent genes closely related to electrical signal transduction and muscle contraction. Interestingly, convergent amino acid substitutions indeed occurred in the *scn4aa* (sodium channel, voltage-gated, type IV, alpha, a) gene in all three electric fish lineages considered in our study; the gene contained a convergent mutated and unique site L1970M in nine focal electric focal fish species from three independent lineages. The *scn4aa* gene apparently experienced strong positive selection in Mormyroidea and Gymnotiformes as reported previously, and this gene is involved in electrical communication (Arnegard et al. 2010). The *scn4aa* gene was also reported by Lamanna et al. (2015) to be involved with ion channel transport and was differentially expressed between EO and SM (skeletal muscle). As for *parp1*, the convergent site was E879D, and the amino acid D uniquely occurs in three electric fish lineages. *Parp1* is a DNA damage-activated enzyme that is associated with multiple DNA repair pathways, especially in response to oxidative stress generated from skeletal muscle contraction (McCrudden et al. 2015; Olah et al. 2015). As for *scamp2*, the convergent amino acid substitution is V252I and was unique to three electric fish lineages. *Scamp2* plays a vital role in neuronal functions and is involved with neurodegeneration (Zheng et al. 2014). Collectively, our genome-wide analysis indicates that natural selection has acted on three electric fish lineages to generate EOs. The overlap between convergent genes and identified PSGs in electric fish lineages (> 10%) may suggest that they have undergone co-occurrence of strong selection and molecular evolution during adaptation to turbid rivers via their abilities to generate electric fields.

## Conclusions

To understand the convergent evolution of electric fishes, we utilized transcriptomic data of the EOs and SMs of nine electric fish species from three independent lineages to search for the signals of convergence. Patterns of evolutionary rates exhibited more rapid genome-wide evolution at the protein level in comparison to the nonelectric fish controls, thereby enabling rapid plasticity and adaptation. A subset of 702 PSGs was putatively associated with electrogenesis. Convergent amino acid substitutions were widespread throughout the electric fish lineages. A subset of convergent genes evolving under positive selection was identified as adaptively convergent genes, and these were putatively associated with electrical and muscular activities, especially *scn4aa* (a voltage-gated sodium channel gene). These results provide valuable insights into the genetic basis for the evolution of electric organs during millions of years of independent evolution.

## Author Contributions

L.Y. conceived this project. Y.W. designed the project. L.Y. performed the computational analyses. Y.W. wrote the paper. L.Y. revised the paper.

## Funding

This work was supported by grants from the National Natural Science Foundation of China (31702016) to Y.W. and from the National Natural Science Foundation of China (31972866) to L.Y.

## Supplementary Material

evab038_Supplementary_Data
